# Oral Administration of *Clostridium butyricum* GKB7 Ameliorates Signs of Osteoarthritis in Rats

**DOI:** 10.3390/cells11142169

**Published:** 2022-07-11

**Authors:** Sunny Li-Yun Chang, Yen-You Lin, Shan-Chi Liu, You-Shan Tsai, Shih-Wei Lin, Yen-Lien Chen, Chin-Chu Chen, Chih-Yuan Ko, Hsien-Te Chen, Wei-Cheng Chen, Chih-Hsin Tang

**Affiliations:** 1 Graduate Institute of Biomedical Science, China Medical University, Taichung 404333, Taiwan; liyunchang@mail.cmu.edu.tw (S.L.-Y.C.); d14333@mail.cmuh.org.tw (C.-Y.K.); 2 School of Medicine, China Medical University, Taichung 404333, Taiwan; chas6119@gmail.com; 3 Department of Medical Education and Research, Beigang Hospital, China Medical University, Yunlin 651012, Taiwan; sdsaw.tw@yahoo.com.tw; 4 Biotech Research Institute, Grape King Bio Ltd., Taoyuan 325002, Taiwan; youshan.tsai@grapeking.com.tw (Y.-S.T.); wei.lin@grapeking.com.tw (S.-W.L.); lan.chen@grapeking.com.tw (Y.-L.C.); 5 Institute of Food Science and Technology, National Taiwan University, Taipei 106617, Taiwan; gkbioeng@grapeking.com.tw; 6 Department of Food Science, Nutrition and Nutraceutical Biotechnology, Shih Chien University, Taipei 104036, Taiwan; 7 Department of Bioscience Technology, Chung Yuan Christian University, Taoyuan 320314, Taiwan; 8 Department of Orthopedic Surgery, China Medical University Hospital, Taichung 404333, Taiwan; bonekid1@gmail.com; 9 Department of Sports Medicine, College of Health Care, China Medical University, Taichung 404333, Taiwan; 10 Department of Medicine, MacKay Medical College, New Taipei 25245, Taiwan; 11 Division of Sports Medicine & Surgery, Department of Orthopedic Surgery, MacKay Memorial Hospital, Taipei 104217, Taiwan; 12 Chinese Medicine Research Center, China Medical University, Taichung 404333, Taiwan; 13 Department of Biotechnology, College of Health Science, Asia University, Taichung 40354, Taiwan

**Keywords:** *Clostridium butyricum* GKB7, osteoarthritis, anterior cruciate ligament transection, in vivo, interleukin 1 beta, tumor necrosis factor alpha

## Abstract

Osteoarthritis (OA) is a degenerative and painful inflammatory joint disease affecting the cartilage, bone, and synovial membranes, without any effective treatment that targets the underlying mechanisms of OA. Our study evaluated the therapeutic effects of a live probiotic strain, *Clostridium butyricum* GKB7, administered for 6 weeks to rats with knee OA (KOA) induced by anterior cruciate ligament transection (ACLT) of the right knee. All rats underwent weekly weight-bearing behavioral testing and body weight measurements. At 6 weeks, all rats were sacrificed, and the right hind knees were collected for micro-computed tomography imaging and histopathological and immunohistochemical analyses. Compared with rats in the ACLT-only group, ACLT rats administered the probiotic exhibited dramatic improvements in pain-related behavior from postoperative week 2, had significantly less osseous and cartilaginous damage at week 6, and significantly lower levels of the inflammatory markers interleukin 1 beta (IL-1β) and tumor necrosis factor alpha (TNF-α) in cartilage and synovium sections. *C. butyricum* GKB7 appeared to slow or even reverse OA progression and is worth investigating as a novel therapeutic for OA.

## 1. Introduction

Osteoarthritis (OA) is a chronic degenerative inflammatory joint disease in which patients experience gradual degradation of the cartilage, subchondral remodeling, and inflammation of the synovial tissue [1,2]. OA is extremely common, affecting one in three people aged over 65 years globally [3], and also expensive, with medical expenditure in the USA estimated to be as high as USD 303 billion in 2013, when calculations included both lost income and medical expenses [4]. The pathogenesis of OA is multifactorial, involving various mechanical, inflammatory, immunological, and metabolic components [5,6,7]. As yet, no approved medications exist that can target the underlying mechanisms of OA pathogenesis and thus slow or reverse its progression [7]. OA treatments, therefore, focus essentially on symptom palliation, with analgesics being a mainstay of treatment. However, the pain relief from paracetamol or opioids is often limited, while nonsteroidal anti-inflammatory drugs (NSAIDs) and opioids are contraindicated in many patients due to well-known side effects [8,9]. Patients with unmanageable symptoms commonly undergo joint replacement, which is an invasive option [8]. Therapeutic agents are greatly needed that target the underlying pathological mechanisms of OA, to prevent or slow its progression.

The gut microbiota ecosystem includes bacteria, viruses, fungi, bacteriophages, and parasites residing in the gastrointestinal tract from the mouth to the anus. The microbiota is involved in many important activities including host immune functioning, regulation of intestinal permeability, nutrient absorption, pathogen resistance, neurotransmitter synthesis and secretion, and metabolic homeostasis [10]. Gut dysbiosis, or disturbance of normal gut microflora, is increasingly recognized as a key factor in the development of OA [11,12], causing permeability of the gut mucosa and disruption of metabolic homeostasis, the generation of systemic inflammation, increasing collagen cleavage in cartilage, and disturbance of the gut-brain axis [11,13]. Amongst the many therapeutic strategies aimed at restoring the gut microbiota ecosystem is the use of probiotics, consisting of a single or combination of bacterial strains [14,15,16,17]. This strategy holds promise for OA.

In this study, we identified a specific *Clostridium butyricum* GKB7 strain that ameliorated OA symptoms in rats with knee OA (KOA) induced by anterior cruciate ligament transection (ACLT). *C. butyricum* is an anaerobic, butyric acid-producing bacillus that is commonly found in the environment, colonizing ~10–20% of adult guts and it has also been found in infant guts [18]. Non-toxigenic strains of *C. butyricum* are already in use as probiotic supplements for improving or preventing gastrointestinal infections, as well as various pathologies including irritable bowel syndrome, inflammatory bowel disease, colorectal cancer, metabolic disorders, multiple sclerosis, systemic lupus erythematosus, and neurodegenerative diseases [18,19,20,21,22]. This study sought to determine the effects of *C. butyricum* GKB7 in rats with ACLT-induced KOA. The findings revealed noticeably fewer weight-bearing deficits, decreased bone loss, and less staining in cartilage and synovial tissue of proinflammatory cytokines interleukin 1 beta (IL-1β) and tumor necrosis factor alpha (TNF-α) in the rats with ACLT-induced KOA that commenced oral *C. butyricum* GKB7 soon after surgery, compared with ACLT-only rats.

## 2. Materials and Methods

### 2.1. Bacterial Strain and Culture Conditions

*C. butyricum* GKB7 was isolated from healthy Taiwanese feces. For seed culture, the bacterial was grown on reinforced clostridial medium (RCM, Merck, Darmstadt, Germany) at 37 °C in an anaerobic chamber under a controlled atmosphere (80% N_2_, 10% H_2_, and 10% CO_2_) for 24 h. To prepare the *C. butyricum* GKB7 spores, 0.1% of the seed culture was scaled-up in a 50 L bioreactor in a culture medium containing 5% glucose, 1.5% yeast extract, 0.5% peptone, 0.1% MgSO_4_, 0.2% K_2_HPO_4_, 0.1% sodium acetate, and 0.2% NaCl for 24 h and centrifuged to harvest the bacterial spores. After 2 washes in reverse osmosis (RO) water, the pellets were freeze-dried and stored at −20 °C until use. The freeze-dried powder contained approximately 5.5 × 10^7^ CFU/g bacterial spores. The dosage applied in this study was 100 mg/kg of body weight per day.

### 2.2. OA Protocol

The 8-week-old Sprague Dawley (SD) rats (300–350 g) used in this study were purchased from the National Laboratory Animal Center in Taipei and maintained in an animal center in China Medical University (CMU), following the university guidelines of its Animal Care Committee. All experimental animal protocols were approved by the CMU Animal Research Ethics Committee (Approval No. CMUIACUC-2021-291). The induction of OA by anterior cruciate ligament transection (ACLT) was performed as previously described [23,24,25]. As described in our recent publications [24,25], we followed an established protocol to induce OA in rats, in which they receive once-weekly injections into the ACLT-operated knee for 6 consecutive weeks [26]. As our previous studies have demonstrated, this protocol results in OA pathological lesions that mainly include synovial inflammation, cartilage degradation, and easily observable subchondral bone lesions [24,25]. We therefore followed the same protocol to evaluate the effects of *C. butyricum* GKB7 in this study. Briefly, the rats were anesthetized with Zoletil 50^®^ (125:125 mg of tiletamine hydrochloride and zolazepam hydrochloride) before undergoing arthrotomy to expose the right knee joint, in which the ACL was cut by micro-scissors using surgical loupes. ACLT success was confirmed by the anterior drawer test. Controls (*n* = 6) underwent arthrotomy without transection of the ACL [27,28]. Starting 2 days after surgery, the daily feed was supplemented by 1 mL RO water in all 3 study groups. *C. butyricum* GKB7 (100 mg/kg) spores were added to the RO water for 6 weeks in the ACLT + *C. butyricum* GKB7 group (*n* = 8), but not to the RO water of the ACLT-only rats (*n* = 6). To confirm that the physical conditions of the experimental rats were not affected by oral administration of *C. butyricum* GKB7 or ACLT-induced KOA, body weights were measured on the day prior to the surgery and every 7 days post-surgery for 6 weeks, before being sacrificed for tissue analysis.

### 2.3. Behavioral Testing

The static weight-bearing incapacitance test (Bioseb, Paris, France) evaluated spontaneous pain after ACLT, as previously described [29,30]. The left and right hind limbs were placed on separate sensor plates to measure between-limb differences in dynamic weight bearing (expressed as grams) over a 10-s period. The mean score of 3 consecutive measurements was recorded for each animal on every test day. To avoid any interference from differences in weights between animals, the results are expressed as a percentage of body weight.

### 2.4. Micro-CT Analysis

Micro-CT analysis was performed at 6 weeks after ACLT surgery. The rats were sacrificed by CO_2_ on day 49 before surgically removing the right lower extremity from each rat. After removing skin and muscle tissue, the intact knee joint was fixed with 4% formaldehyde and then 70% ethanol at room temperature, as previously described [27,29]. The knee joints were scanned at the voxel resolution of 10.5 µm of micro-focus mode using a SkyScan 2211 micro-CT system (Bruker, Kontich, Belgium) using micro-CT cameras that scanned over 180° of rotation with a voltage of 70 kVp, a current of 290 µA, and a 0.5 mm aluminum filter to prevent beam-hardening artifacts. Image reconstruction of coronal and transverse images, ring artifact, and beam-hardening corrections were performed using InstaRecon^®^ software (Bruker micro-CT, Kontich, Belgium). Reconstructed cross-sections were reorientated and 59 slices (0.5 mm) were selected, then manual regions of interest (ROI) were drawn of an irregular contour in the subchondral trabecular bone region for the medial tibial plateau. Images were analyzed for thresholding, regions of interest, bone morphometric analysis, and bone mineral density (BMD), bone mineral content (BMC), bone volume/total volume (BV/TV), bone surface/total volume (BS/TV), trabecular number (Tb.N), trabecular thickness (Tb.Th), and trabecular separation (Tb.Sp) date using Bruker micro-CT software (CTAn, version 1.7.1, Bruker, Kontich, Belgium), as previously detailed [27,29].

### 2.5. Histopathological Analysis

After undergoing micro-CT scanning, the knee joint tissues were fixed with 4% paraformaldehyde in phosphate-buffered saline (PBS) at 37 °C for 24 h then decalcified in 10% EDTA at 4 °C for 2 weeks. After dehydration with increasingly higher concentrations of ethanol (from 70% to 100%), the joint tissues were embedded into paraffin blocks and cut into slices of 5 µm thicknesses. Hematoxylin and eosin (H&E) and Safranin-O/Fast Green staining analyzed histopathological changes in OA tissue under an optical microscope, as previously described [31,32]. Changes in structural cartilage in the central weight-bearing area of the medial tibial plateau were evaluated by the Osteoarthritis Research Society International (OARSI) histopathology grading system [32,33]. Damage was graded from 0 to 6 representing the depth of OA progression into the cartilage of the sagittal sections and the stage of damage was defined as the horizontal extent of cartilage involvement from 0 to 4. The final score combined grade and stage (score range 0–24). Observer bias was avoided by having two independent assessors perform the scoring of changes in the knee joint, as in previous studies [32,34].

### 2.6. Immunohistochemical Analysis

For analysis of IL-1β and TNF-α expression, the tissue sections were incubated with 3% hydrogen peroxide to block endogenous peroxidase activity then incubated again with 3% bovine serum albumin in PBS to reduce the non-specific background. After incubation with primary antibodies against IL-1β (1:200; MAB601, R&D systems, Minneapolis, MN, USA) or TNF-α (1:200; A11534, ABclonal, Woburn, MA, USA) at 4 °C overnight, followed by incubation with secondary antibody (1:200) at room temperature for 1 h, the sections were stained with diaminobenzidine and observed under a light microscope, as previously described [35,36,37]. The intensities of the immunoreactive signals were scored from 1 to 5 (from weak to strong) for positive expression by two independent assessors who were blinded to the treatment groups to minimize observer bias.

### 2.7. Statistical Analysis

Statistical calculations were performed using PRISM 5.0 software (GraphPad, San Diego, CA, USA) and all values are expressed as the mean ± standard deviation (S.D.). The paired sample *t*-test was selected to compare results from two groups. One-way ANOVA followed by Bonferroni post hoc testing for multiple comparisons was used to analyze statistical data from more than two groups. A *p*-value of less than 0.05 was considered to be statistically significant.

## 3. Results

### 3.1. Oral C. butyricum GKB7 Does Not Affect Body Weight

**Figure 1:**
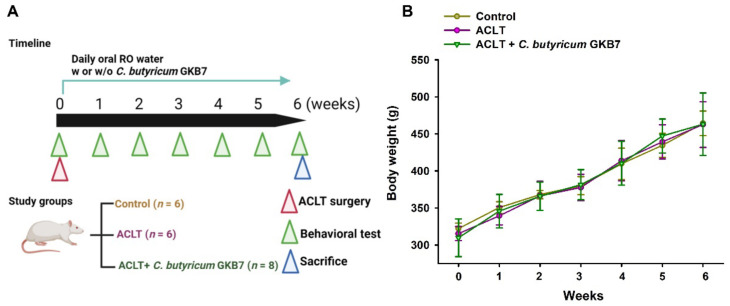
(**A**) Experimental design. (**B**) Body weights throughout the experimental period. Although the body weights of all rats increased during the experiment, the weekly measurements did not exhibit any significant variations between the study groups.

After habituation for 3–5 days in the animal center, the rats were randomly assigned to one of three study groups: arthrotomy without ACLT (controls); ACLT only; or ACLT + oral *C. butyricum* GKB7. Body weights were monitored the day before surgery and every week thereafter until the rats were sacrificed for analysis. The body weights of all groups gradually increased during the experimental period, with no significant between-group differences (Figure 1), which implies that *C. butyricum* GKB7 had no toxic side effects.

### 3.2. Oral C. butyricum GKB7 Ameliorates OA Pain

**Figure 2:**
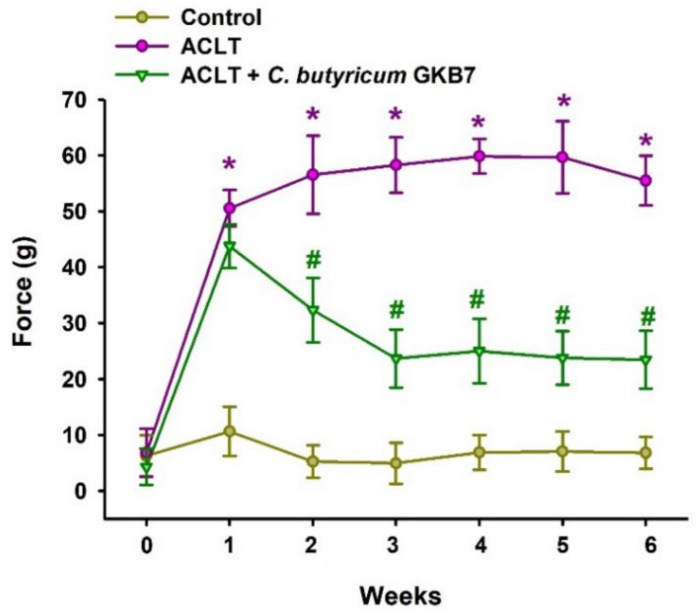
Oral *C. butyricum* GKB7 improves weight-bearing asymmetry. Behavioral testing for pain suggested that by postoperative week 2, rats in the ACLT + *C. butyricum* GKB7 group were in less pain than rats in the ACLT-only group. * *p* < 0.05 ACLT-only vs. controls; # *p* < 0.05 ACLT-only vs. ACLT + *C. butyricum* GKB7.

We performed the static weight-bearing incapacitance test the day before surgery and every week during the experimental period to evaluate the therapeutic effect of *C. butyricum* GKB7 on OA pain [29]. ACLT-only rats exhibited severe asymmetry in weight-bearing posture from the first week that worsened throughout the experimental period, whereas the ACLT + *C. butyricum* GKB7 group showed dramatic improvements in pain-related behavior beginning the second week postoperatively, which improved to such an extent that by weeks 3–6 the asymmetry in weight-bearing behavior was only one-third that of the ACLT-only group (Figure 2). In the first week postoperatively, there was no significant difference between the ACLT-only and ACLT + *C. butyricum* GKB7 groups. This suggests that *C. butyricum* GKB7 may be able to efficiently treat OA-related pain and that its onset of action requires at least 7–14 days with optimal effects reached after 21 days of continuous oral administration in rats.

### 3.3. Oral C. butyricum GKB7 Protects against or Repairs Osseous Damage in OA

**Figure 3:**
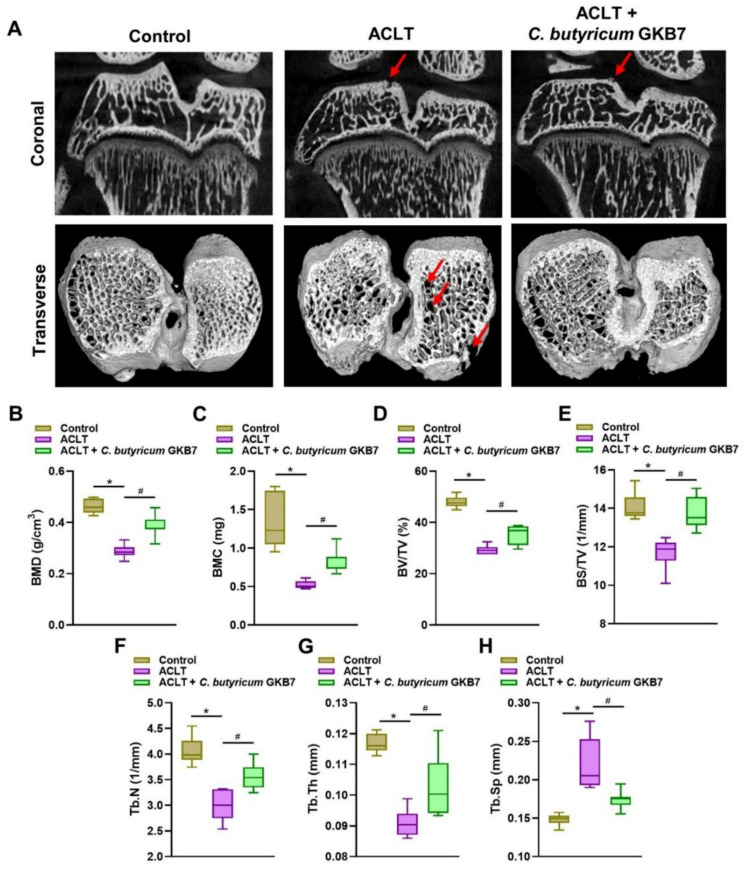
Oral *C. butyricum* GKB7 ameliorates osseous damage in the ACLT-induced OA knee joint. (**A**) Micro-CT images of the right knee joints of controls, ACLT-only and ACLT + *C. butyricum* GKB7-treated rats. Red arrow indicates the bone loss. (**B**–**H**) Quantitative analyses of the bone mineral density (BMD, B), bone mineral content (BMC, C), bone volume/total volume (BV/TV, D), bone surface/total volume (BS/TV, E), trabecular number (Tb.N, F), trabecular thickness (Tb.Th, G), and trabecular separation (Tb.Sp, H) data in all study groups. * *p* < 0.05 ACLT-only vs. controls; # *p* < 0.05 ACLT-only vs. ACLT + *C. butyricum* GKB7.

Six weeks after ACLT surgery, micro-CT analysis showed significant bone destruction in ACLT-only rats compared with controls, confirming the procedure’s ability to create an OA model (Figure 3A). In contrast, the ACLT + *C. butyricum* GKB7 group had significantly less bony damage (Figure 3A). Micro-CT images indicated significant improvements in bone architecture in rats administered *C. butyricum* GKB7 compared with ACLT alone, as determined by BMD, BMC, BV/TV, BS/TV, Tb.N, Tb.Th, and Tb.Sp data (Figure 3B–H).

### 3.4. Oral C. butyricum GKB7 Protects against ACLT-Induced Articular Cartilage Damage

**Figure 4:**
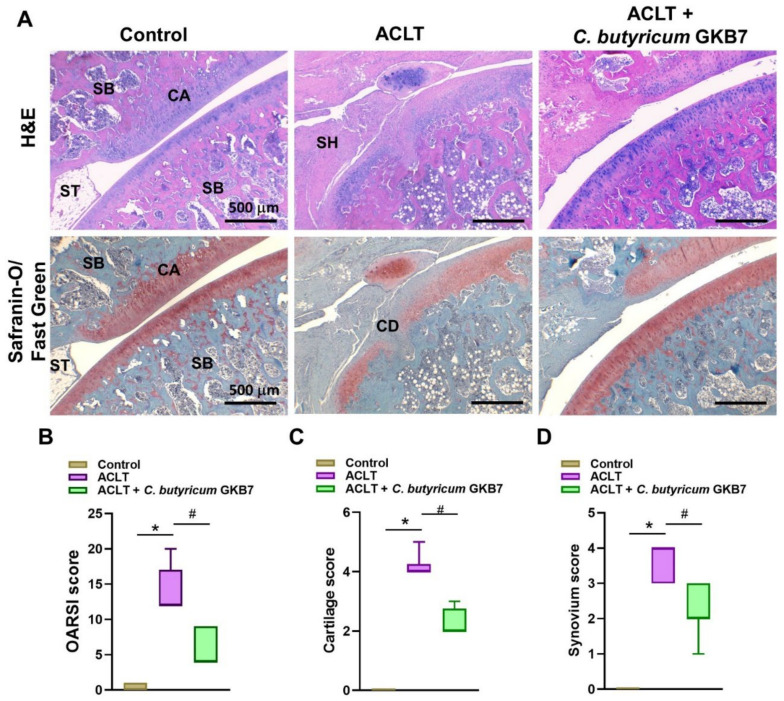
Oral *C. butyricum* GKB7 ameliorates articular cartilage damage. (**A**) Histopathological analysis of right knee articular cartilage specimens stained with H&E and Safranin-O/Fast Green. Less severe cartilage damage (CD) was observed in the ACLT + *C. butyricum* GKB7 group compared with the ACLT-only group. (**B**–**D**) Assessment of osteoarthritic cartilage histopathology by OARSI total scores (**B**), cartilage degeneration scores (**C**), and synovial membrane inflammation scores (**D**). Abbreviations: ST, synovial tissue; CA, cartilage; SB, subchondral bone; SH, synovial hyperplasia. * *p* < 0.05 ACLT-only vs. controls; # *p* < 0.05 ACLT-only vs. ACLT + *C. butyricum* GKB7.

H&E and Safranin-O/Fast Green staining revealed degenerative changes in articular cartilage and synovial lining hyperplasia (CD and SH, respectively) in the ACLT knee specimens (Figure 4A). Fewer pathological changes in cartilage tissue and less synovial tissue hyperplasia were found in samples of ACLT rats treated with *C. butyricum* GKB7 compared with ACLT-only rats.

### 3.5. Oral C. butyricum GKB7 Downregulates TNF-α and IL-1β Expression in OA Cartilage and Synovium

**Figure 5:**
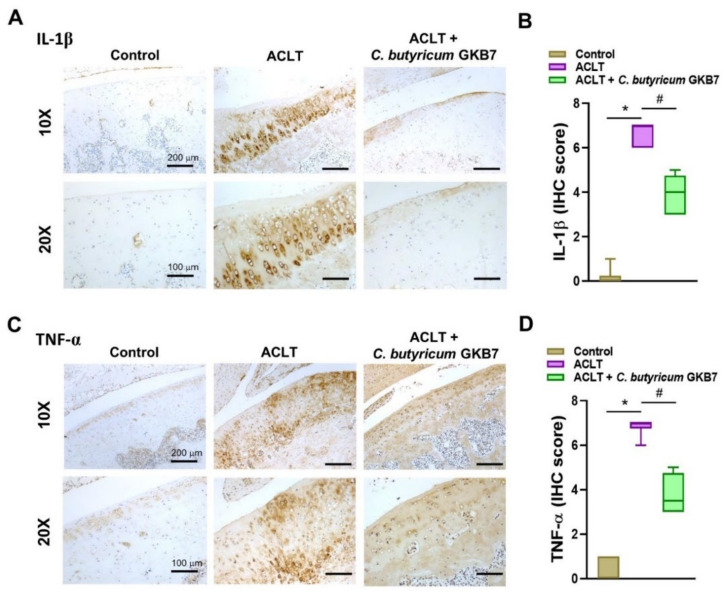
Oral *C. butyricum* GKB7 downregulates IL-1β and TNF-α in OA articular cartilage. Immunohistochemistry analysis and scoring of IL-1β (**A**,**B**) and TNF-α (**C**,**D**) in right knee joint cartilage. * *p* < 0.05 ACLT-only vs. controls; # *p* < 0.05 ACLT-only vs. ACLT + *C. butyricum* GKB7.

**Figure 6:**
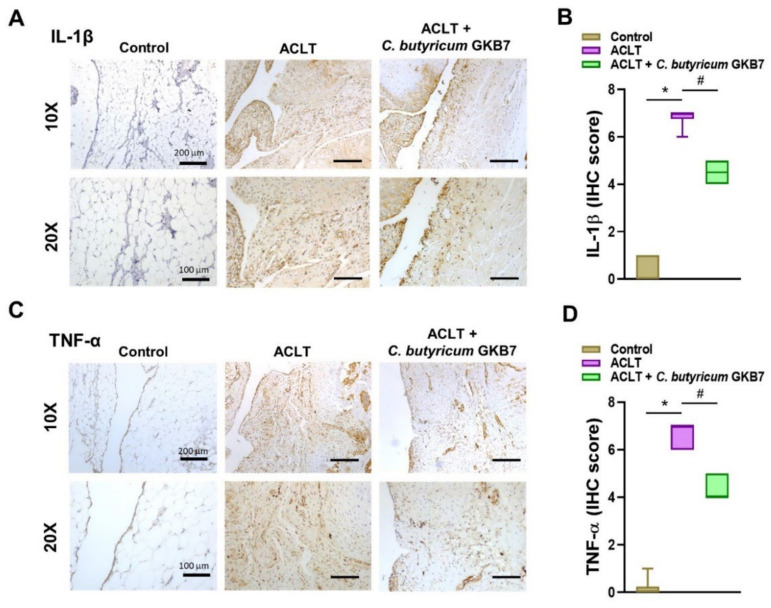
Oral *C. butyricum* GKB7 downregulates IL-1β and TNF-α in OA synovium. (**A**,**B**) Immunohistochemistry analysis and scoring of levels of IL-1β and (**C**,**D**) TNF-α expression in right knee joint synovium. * *p* < 0.05 ACLT-only vs. controls; # *p* < 0.05 ACLT-only vs. ACLT + *C. butyricum* GKB7.

Proinflammatory cytokines IL-1β and TNF-α play vital roles in OA, particularly during the early symptomatic stages [18,19]. In this study, levels of IL-1β and TNF-α expression were dramatically upregulated in the chondrocytes of OA joint cartilage, whereas there was little or no cytokine expression in control cartilage (Figure 5). Cartilage and synovium from the ACLT + *C. butyricum* GKB7 group exhibited moderate levels of IL-1β and TNF-α expression and no significant synovial hyperplasia (Figure 6).

## 4. Discussion

OA was originally thought to be a degenerative disease caused by mechanical stress, particularly on load-bearing joints (knees and hips). It has now become clear that systemic inflammation, immune dysfunction, disorders in metabolism, and dysregulation of the gut microbiome are important factors in OA pathology [11,12]. Probiotics are known to be capable of ameliorating gut dysbiosis and multiple probiotic strains have demonstrated therapeutic effects in OA. For instance, 28 days of orally administered *Lactobacillus rhamnosus* after OA was induced in rats by monosodium iodoacetate (MIA) injection reduced pain severity and cartilage damage, and also increased levels of anabolic factors and chondrogenic transcription factors, with accompanying reductions in intestinal damage and inflammation [38]. In another study involving MIA-induced OA rats, the severity of OA pain, cartilage damage, and the extent of lymphocyte infiltration were all reduced by a greater extent when oral *L. casei* was co-administered with type II collagen and glucosamine, compared with administration of *L. casei* or glucosamine alone [39]. That study also found that the combination of *L. casei*, type II collagen and glucosamine was associated with reductions in proinflammatory cytokines IL-1β, IL-2, IL-6, IL-12, IL-17, TNF-α, and interferon-γ, as well as collagen catabolic matrix metalloproteinases (MMP1, MMP3, and MMP13), while increases were observed in levels of anti-inflammatory IL-4 and IL-10 cytokines [39]. Similar results have been observed in humans given *L. casei* and in animal studies involving *L. acidophilus*, *Bifidobacterium longum* CBi0703, and *Streptococcus thermophilus* [12,38].

In this study, we demonstrate that a newly identified strain of *C. butyricum* (GKB7) ameliorates the progression of OA symptoms including joint pain, cartilage damage, and synovial hyperplasia. To our knowledge, our study is the first to explore the effect of the GKB7 strain of *C. butyricum* in OA and to do so as a live, rather than a heat-treated, preparation. We examined differences in weight distribution between each rat’s normal vs ACLT-treated knee as a proxy of OA symptom severity, as pain would cause a shift in weight to the unaffected limb [29]. In week 1, there was no statistically significant difference in weight distribution between the ACLT-only and ACLT + *C. butyricum* GKB7 groups, but by week 2 the probiotic-treated rats were demonstrating marked improvements in symptoms that plateaued in week 3. *C. butyricum* GKB7 evidently has potential for both slowing OA progression and ameliorating symptoms.

In our study, the effect of *C. butyricum* GKB7 on OA was achieved without changes in body weight between experimental groups, so it was unlikely to be due to a reduction in mechanical joint loading. High body mass index is a risk factor for OA, not just because of excessive mechanical loading, but also because of the systemic inflammation associated with obesity [40,41]. Our study suggests that *C. butyricum* GKB7 improves OA pathology without having to affect obesity-related systemic inflammation. The normal body weights of the rats in this study also indicate that *C. butyricum* GKB7 is less likely to have toxic side effects that might be evidenced by weight loss. In addition, our findings imply that *C. butyricum* does not have anti-obesogenic weight loss properties that have been seen with other probiotic strains such as *L. casei*, *L. rhamnosus,* and *L. gasseri* [42].

*C. butyricum* has been used as a probiotic for the prevention or treatment of antibiotic-associated diarrhea, pouchitis, ulcerative colitis, irritable bowel syndrome, vascular dementia, acute liver injury, colorectal cancer, type 1 and 2 diabetes, acute pancreatitis, and necrotic enteritis [18,40,43]. One of the end products of *C. butyricum* fermentation is butyrate, a short-chain fatty acid that is the main energy source of colonic bacteria, which has anti-inflammatory, microbiome modulating effects and gut membrane protective effects [18]. By suppressing effector T cells, butyrate dampens inflammatory activation and, thus, restores immune homeostasis [18]. The production of butyrate may stimulate the survival of other beneficial butyrate-producing bacteria [18]. *C. butyricum* also regulates gut barrier permeability by increasing colonic mucosal wall thickness and butyrate can specifically increase mucin production, which together prevent the leakage of microbes and their toxins into the circulation and ultimately reduce systemic inflammation [18]. While butyrate is not the only active metabolite of *C. butyricum* that explains its mechanism of action, it certainly is a critical one.

The effects of *C. butyricum* in OA have also been reported by other researchers who administered a tyndalized, sterilized *C.* *butyricum* IDCC 5101 microorganism (ID-CBT5101; Ildong Pharmaceuticals) to rats with MIA-induced OA [13]. In that study, the rats received either 10^8^ colony-forming units (CFU) per day or 10^10^ CFU/day of *C. butyricum* for 2 weeks prior to MIA induction of OA and then for 4 weeks postoperatively [13]. Both study groups demonstrated marked improvements in MIA-induced OA progression with reductions in systemic inflammation, abnormal bone metabolism, and inappropriate collagen degradation, compared with untreated MIA-injected rats [13]. Moreover, IL-6 and leukotriene B4 inflammatory markers were significantly decreased in both *C. butyricum* treatment groups, although the inflammatory mediator COX-2 was only reduced in the 10^10^ CFU/day group [13], which implies different dosage effects of *C. butyricum* in OA. It will be worth confirming in future studies whether different dosages of our newly identified *C. butyricum* GKB7 strain result in different therapeutic responses.

Sim et al. [13] have also reported bone and cartilage benefits similar to those in our study. Those researchers reported functional weight-bearing measurements showing that although both *C. butyricum* groups had improvements in OA symptoms as early as day 9, the 10^10^ CFU/day dosage was associated with superior pain reduction compared with the 10^8^ CFU/day dosage indicating a dose response that appeared to plateau by day 13 [13]. In our study, we did not see a decline in pain behaviors until after week 1 and this decline continued through week 3. This may indicate that a live culture of *C. butyricum* has a delayed onset but longer duration of action than a heat-treated preparation, or it may be explained by the different strains of *C. butyricum* used in our study and that by Sim et al. [13]. The variation in onset and duration may also be attributed to different concentrations and potencies of *C. butyricum* given in our study compared with the preparation given by Sim et al. [13], making it possible for those researchers to reach the same end points in OA symptom reduction within a shorter time. A limitation of this study is the fact that the underlying mechanisms that lead to the effectiveness of *C. butyricum* GKB7 in experimental OA remain undefined. This is a worthwhile topic for future investigation.

Significant reductions in the inflammatory mediators IL-1β and TNF-α in both cartilage and synovium after *C. butyricum* GKB7 administration partially explain the improved quality and quantity of bone and cartilage structure in rats with ACLT-induced OA. One research group has shown that coloclysis administration of *C. butyricum* ameliorates bone loss induced by alternations in gut microbiota after bariatric surgery [44]. Osteoblasts play a crucial role in OA pathogenesis [45], although they may also slow the progression of OA disease. For instance, treating OA osteoblasts with nacre extract (isolated from oyster shell) restores their mineralization capacity [46]. It would be interesting to determine whether the oral administration of live *C. butyricum* GKB7 affects the function of OA osteoblasts and slows the disease progression. It would also be worth examining in future studies whether different experimental doses of the *C. butyricum* GKB7 strain results in different therapeutic responses; this study only examined a single dose of *C. butyricum* GKB7.

## 5. Conclusions

In conclusion, our findings suggest that oral administration of *C. butyricum* GKB7 slows the progression of ACLT-induced OA. This treatment downregulated critical proinflammatory markers in OA cartilage and synovium, improved weight-bearing asymmetry without adversely affecting body weight, and decreased bone loss in rats with ACLT-induced OA. This research offers exciting possibilities for clinical medicinal options for OA treatment.

## Data Availability

The raw data for this study are available from the corresponding authors.
